# Proteinuria is a risk factor for acute kidney injury after cardiac surgery in patients with stages 3–4 chronic kidney disease: a case control study

**DOI:** 10.1186/s12872-023-03102-4

**Published:** 2023-02-10

**Authors:** Wuhua Jiang, Zhihong Chen, Jiarui Xu, Zhe Luo, Jie Teng, Xiaoqiang Ding, Shuan Zhao, Xialian Xu

**Affiliations:** 1 Department of Nephrology, Zhongshan Hospital (Xiamen), Fudan University, Xiamen, China; 2 Department of Nephrology, Zhongshan Hospital, Fudan University, No 180 Fenglin Road, Shanghai, 200032 China; 3 Shanghai Medical Center of Kidney, Shanghai, China; 4 Shanghai Key Laboratory of Kidney and Blood Purification, Shanghai, China; 5 Department of Critical Care, Zhongshan Hospital, Fudan University, Shanghai, China

**Keywords:** Proteinuria, Acute kidney injury, Cardiac surgery

## Abstract

**Background:**

Acute kidney injury (AKI) is a common complication after cardiac surgery, and preoperative renal dysfunction is an important risk factor. Proteinuria indicates renal structural damage, but there are few studies on proteinuria and the risk of AKI after cardiac surgery in patients with renal dysfunction. This study aimed to elucidate whether proteinuria can predict AKI after cardiac surgery in patients with renal dysfunction.

**Methods:**

Patients with stages 3–4 chronic kidney disease (CKD) who underwent cardiac surgery were included in this retrospective study. AKI was defined according to the KDIGO criteria. The association between proteinuria and AKI in patients with CKD stages 3–4 was investigated.

**Results:**

The incidence of AKI in the entire cohort (n = 1546) was 53.55%. The in-hospital mortality of patients with was higher than patients without AKI (AKI vs. no AKI, 4.7 vs. 0.8%, *P* < 0.001). Multivariate logistic regression analysis showed that proteinuria was an independent risk factor for AKI (trace to 1+ OR 2.37; 2+ –3+ OR 5.16) and AKI requiring renal replacement therapy (AKI-RRT) (trace to 1+ OR 3.64; 2+–3+ OR 5.71). Mild proteinuria (trace to 1+ OR 2.59) was also an independent risk factor for in-hospital death. In patients with diabetes mellitus, mild proteinuria (OR 1.925), instead of severe proteinuria (2–3+), was a risk factor of AKI in patients with kidney dysfunction and diabetes.

**Conclusions:**

In the population of patients with renal dysfunction, the incidence of AKI was high, which significantly compromised renal and overall prognosis. As a simple and inexpensive routine test, preoperative proteinuria still has value in predicting AKI in patients with impaired renal function.

**Supplementary Information:**

The online version contains supplementary material available at 10.1186/s12872-023-03102-4.

## Background

AKI is a common complication after cardiac surgery. Epidemiological studies have shown that AKI is closely associated with high mortality, length of hospital stay, hospital stay cost, and poor long-term prognosis [1–3]. With the continuous development of cardiac surgery technology, more and more patients with kidney disease are eligible for surgery. The incidence of chronic kidney disease is increasing worldwide [4]. Studies have shown that preoperative renal dysfunction is an important risk factor and susceptibility factor for AKI after cardiac surgery [5]. AKI is more drastic, more serious and has a worse prognosis in patients with renal dysfunction. Therefore, early identification of AKI in patients with renal dysfunction and timely intervention to improve prognosis has become a research focus.

Proteinuria is a marker of renal structural damage. Epidemiological studies in the past decade have suggested the predictive role of proteinuria in AKI after cardiac surgery [6–8]. However, some patients with renal dysfunction also have proteinuria. Compared with patients with normal or mildly impaired renal function, patients with CKD stages 3–4 are susceptible to perioperative risk factors during cardiac surgery and prone to develop AKI. Biomarkers for predicting AKI or poor outcome in patients with CKD have been reported [9, 10]. As a convenient and inexpensive routine test, it would be inspiring if proteinuria could be of value in predicting AKI and outcome in patients with stages 3–4 CKD. This study will investigate the predictive role of preoperative proteinuria on AKI after cardiac surgery in patients with impaired renal function.

## Methods

### Patients and inclusion/exclusion criteria

We enrolled adult patients suffered from renal dysfunction (15 ≤ eGFR < 60 ml/min/1.73m^2^) who underwent valve, coronary artery bypass surgery (CABG) or combined surgery from January 2017 to December 2020. Among the exclusion criteria are: (1) Patients with AKI defined according to the KDIGO criteria before surgery; (2) History of renal replacement therapy or kidney transplantation; (3) Data on the medical history are incomplete; (4) Patients who were deceased ≤ 24 h after they were admitted to the ICU; (5) Patients taking nephrotoxin (e.g. aminoglycosides, non-steroidal anti-inflammatory drugs) within 2 weeks before surgery; (6) Patients undergoing minimally invasive valve surgery were not included in this study to avoid the adverse effects of contrast media. The Institutional Ethical Committee of Zhongshan Hospital granted permission for study design and data collection, and due to the non-interventional design, informed consent was not required. The study was conducted in accordance with the Helsinki Declaration.

### Design

The study was retrospective observational in nature. Detailed clinical information such as demographics, underlying heart disease, comorbidities, preoperative heart function, preoperative laboratory examination (blood test), urine routine test, medication history, surgery type, cardiopulmonary bypass (CPB) duration, and postoperative care, urine output, length of hospital stay, ICU stay, and mortality were accessed through the electronic medical records. Data were followed up until discharge or death. AKI was the primary outcome. The patients were classified with the occurrence and severity of proteinuria. An investigation of the risk factors of postoperative AKI in chronic kidney disease patients was conducted.

Additionally, in-hospital death and postoperative AKI requiring renal replacement therapy (RRT) were assessed as secondary outcomes. The decision to initiate dialysis was at the discretion of the consulting nephrologist after metabolic abnormalities (acidosis, hyperkalemia, anuria), and fluid overload were identified.

Based on patient characteristics and the latest serum creatinine measurement prior to surgery, an estimate of glomerular filtration rate (eGFR) was derived by the CKD-EPI formula. It was standard of care to measure SCr daily in the postoperative ICU.

The Urine samples were collected early in the morning within two days prior to surgery and proteinuria was measured with a dipstick. To classify the severity of proteinuria, we defined negative as “no proteinuria,” trace to 1+ as “mild proteinuria,” and 2+ to 3+ as “severe proteinuria.” The test strips were measured by an automatic dipstick autoanalyzer (AUTION MAX, AX-4030; ARKRAY Inc., Kyoto, Japan).

The statistical analysis was performed using IBM SPSS 25.0 (Armonk, NY, USA). Values are presented as the mean ± standard deviation for data that were normally distributed or median and interquartile range for data that were not normally distributed for continuous variables and number (%) for categorical variables. All the data were checked for normality and homogeneity of variance using the Kolmogorov–Smirnov test. *P* values were derived from the one-way Student t test to determine differences between groups with normally distributed data and Mann–Whitney nonparametric test with other variables for two-group comparisons. Categorical variables were compared using Fisher's exact test or chi-squared test. In order to detect correlations between variables and proteinuria, Pearson correlation was applied. For the determination of the risk factors for AKI, the intraoperative and postoperative characteristics of patients were compared using a univariable logistic regression analysis. The odds ratio (OR) with 95% confidence interval (CI) was calculated for the predictors of AKI. In addition, the multivariable logistic regression analysis was carried out using the stepwise forward selection method for variables with a *P* value of < 0.05 considered predictive for AKI. We also performed a subgroup analysis of patients with diabetes mellitus to investigate the relationship between proteinuria and AKI. For all comparisons, *P* < 0.05 was considered statistically significant.

## Results

### Basic characteristics

A total of 1546 patients were included in the study. According to the degree of proteinuria or not, the patients were classified into three groups, including 249 cases of mild proteinuria (Trace-1+) and 56 cases of moderate and severe proteinuria (2–3+). The proportion of male and diabetic patients with proteinuria was significantly higher than those without proteinuria. Patients with proteinuria had lower left ventricular ejection fraction and hemoglobin, and worse preoperative basic renal function. The proportion of double valve surgery was higher in patients with proteinuria. AKI and in-hospital mortality were significantly higher in patients with proteinuria than in patients without proteinuria (Table 1).

**Table 1:** Perioperative characteristics of the study population

Characteristics	No proteinuria (N = 1241)	Mild proteinuria (Trace-1 +) (N = 249)	Severe proteinuria (2–3 +) (N = 56)	*P*
Demographic data				
Male (%)	697 (56.2)	153 (61.4)	40 (71.4)	0.031
Age (years)	64.25 ± 10.02	61.82 ± 11.99	62.38 ± 10.56	0.002
BMI (kg/m^2^)	23.38 ± 3.17	23.45 ± 3.08	23.04 ± 3.31	0.695
Comorbidities				
Hypertension (%)	643 (51.9)	142 (57.0)	33 (58.9)	0.216
DM (%)	186 (15.0)	68 (27.3)	15 (26.8)	0.001
NYHA grade 3–4 (%)	808 (69.8)	165 (69.3)	37 (67.3)	0.92
LVEF	58 ± 10.43	55.58 ± 10.74	53.59 ± 11.86	0.004
Medication				
Statin	74 (59.71)	162 (65.06)	37 (66.07)	0.205
Renin–angiotensin–aldosterone system inhibitor	615 (49.55)	128 (51.40)	24 (42.85)	0.511
*Primary heart diseases				
Mitral stenosis	373 (30.05)	64 (25.70)	16 (28.57)	0.384
Mitral regurgitation	193 (15.55)	45 (18.07)	10 (17.85)	0.586
Aortic stenosis	125 (10.07)	25 (10.04)	7 (12.5)	0.839
Aortic regurgitation	201 (16.19)	37 (14.85)	7 (12.5)	0.682
Coronary artery disease	504 (40.61)	125 (50.20)	23 (41.07)	0.02
Baseline laboratory indices				
Hemoglobin (g/L)	126.71 ± 17.80	122.58 ± 20.53	122.67 ± 19.90	0.003
Albumin (g/L)	39.57 ± 3.59	38.37 ± 4.32	39.41 ± 3.43	< 0.001
BUN (mmol/L)	9.51 ± 4.76	11.54 ± 5.78	11.43 ± 4.99	< 0.001
Serum creatinine (μmol/L)	129.02 ± 58.58	158.55 ± 76.42	180.88 ± 130.95	< 0.001
eGFR (ml/min/1.73m^2^)	49.16 ± 9.73	42.50 ± 13.06	42.37 ± 14.73	< 0.001
Uric acid(μmol/L)	474.6 ± 153.8	538.6 ± 385.1	508.7 ± 122	< 0.001
Surgery				
Sole valve (%)	737 (59.4)	124 (49.8)	26 (46.4)	0.024
Aortic	154 (12.41)	18 (7.29)	3 (5.35)	0.145
Mitral	335 (26.99)	38 (15.26)	8 (14.28)	0.004
Tricuspid	56 (4.51)	3 (1.20)	2 (3.57)	0.107
Double/Triple	192 (15.47)	65 (26.10)	13 (23.21)	< 0.001
Sole valvuloplasty	97 (7.8)	14 (5.62)	4 (7.14)	0.483
Sole replacement	301 (24.25)	34 (13.65)	7 (12.5)	< 0.001
Valvuloplasty + re-placement	339 (27.31)	76 (30.52)	15 (26.78)	0.579
Sole CABG (%)	426 (34.4)	110 (44.2)	25 (44.7)	0.263
Valve & CABG (%)	78 (6.3)	15 (6.0)	5 (8.9)	0.754
CPB duration (mins)	107.33 ± 67.33	104.75 ± 39.69	108.17 ± 37.7	0.883
Cross-clamp duration (mins)	63.89 ± 51.76	58.66 ± 26.82	67 ± 31.45	0.443
#Postoperative major bleeding	14 (1.12)	3 (1.20)	2 (3.50)	0.268
Prognosis				
AKI	598 (48.2)	181 (72.7)	49 (87.5)	0.001
In-hospital mortality	27 (2.2)	14 (5.6)	4 (7.1)	0.002
Length of ICU stay (h)	46 (22, 90)	50 (23, 120)	81 (24,120)	0.006
Length of hospital stay (days)	14 (11, 19)	15 (11, 22)	13 (10, 20)	0.042

AKI: Acute kidney injury; BMI: Body Mass Index; BUN: Blood Urea Nitrogen; CABG: Coronary artery bypass grafting; CPB: Cardiopulmonary bypass; DM: Diabetes mellitus; eGFR: Estimated glomerular filtration rate, calculated by CKD-EPI formulae; ICU: intensive care unit; LVEF: Left ventricular ejection fraction; NYHA: New York Heart Association

The values are expressed as the median (IQR) and mean ± SD or number (%)

*P* values are the results of unpaired t-test or Mann–Whitney U test for continuous variables, and χ^2^ test or Fisher’s exact test for categorical variables

^*^Diagnosis of primary heart disease was derived from the electronic medical record system, and many patients with multiple heart conditions have several diagnoses

^#^The postoperative major bleeding included chest tube output 400 ml/h or 200 ml/h for 3 h or re-operation after closure of sternotomy for the purpose of controlling bleeding

## Risk factors for AKI development

Logistic regression analysis showed that both mild (OR for trace to 1+ proteinuria = 2.370, 95% CI 1.52–3.70, *P* < 0.001) and severe proteinuria (OR for 2–3+ proteinuria = 5.161, 95% CI 1.12–23.82, *P* = 0.035), decreased preoperative hemoglobin (OR 0.984, 95% CI 0.98–0.99, *P* < 0.001), male gender(OR 1.76 95% CI 1.30–2.39, *P* < 0.001), and increased intraoperative aortic clamping time (OR 0.984, 95% CI 0.98–0.99, *P* < 0.001) were associated with postoperative AKI (Table 2).

**Table 2:** Logistic regression of risk factors for multiple endpoints

	Univariate analysis			Multivariate analysis		
	OR	95%CI	P value	OR	95%CI	*P* value
*AKI*						
Male	1.42	1.16–1.74	0.001	1.76	1.30–2.39	< 0.001
Hypertension	1.40	1.15–1.72	0.001			NS
Diabetes Mellitus	1.11	0.85–1.45	0.432			NS
NYHA grade 3–4 (%)	1.25	1.00–1.57	0.045			NS
*Baseline laboratory indices*						
eGFR (ml/min/1.73m^2^)	0.98	0.98–0.99	0.002			NS
UA (μmol/L)	1.00	1.00–1.01	0.011			NS
Hemoglobin (g/L)	0.98	0.98–0.99	< 0.001	0.984	0.98–0.99	< 0.001
Albumin (g/L)	0.93	0.90–0.95	< 0.001			NS
*Proteinuria*						
Negative	1			1		
Trace-1+	2.86	2.12–3.86	< 0.001	2.370	1.52–3.70	< 0.001
2+–3+	7.52	3.38–16.74	< 0.001	5.161	1.12–23.82	0.035
Valve & CABG	2.61	1.63–4.16	< 0.001			NS
CPB duration (mins)	1.00	1.00–1.01	0.001			NS
Cross-clamp duration (mins)	1.00	1.00–1.01	0.001	1.010	1.00–1.02	< 0.001
*AKI-RRT*						
Diabetes Mellitus	1.528	0.92–2.54	0.102			NS
*Baseline laboratory indices*						
eGFR (ml/min/1.73m^2^)	0.93	0.92–0.95	< 0.001	0.958	0.94–0.98	< 0.001
UA (μmol/L)	1.00	1.00–1.01	< 0.001	1.001	1.00–1.01	0.044
Hemoglobin (g/L)	0.97	0.96–0.99	< 0.001			NS
Albumin (g/L)	0.91	0.86–0.97	0.006			NS
*Proteinuria*						
Negative	1			1		
Trace-1+	5.17	3.20–8.38	< 0.001	3.639	1.99–6.64	< 0.001
2+–3+	11.58	5.90–22.72	< 0.001	5.71	1.88–17.36	0.002
Valve & CABG	3.23	1.73–6.02	< 0.001	4.02	1.89–8.53	< 0.001
*In-hospital death*						
Diabetes Mellitus	1.19	0.57–2.50	0.64			NS
*Baseline laboratory indices*						
eGFR (ml/min/1.73m^2^)	0.96	0.94–0.99	0.01	0.97	0.94–0.99	0.007
Albumin (g/L)	0.92	0.85–0.99	0.04			NS
*Proteinuria*						
Negative	1			1		
Trace-1+	2.68	1.38–5.18	0.003	2.59	1.29–5.23	0.008
2+–3+	3.46	1.16–10.25	0.025			NS
Valve & CABG	3.02	1.26–7.27	0.013			NS

AKI: Acute kidney injury; AKI-RRT: Acute kidney injury requiring renal replacement therapy; CABG: Coronary artery bypass grafting; CI: Confidential interval; CPB: Cardiopulmonary bypass; eGFR: Estimated glomerular filtration rate, calculated by CKD-EPI formulae; NS: Not Significant; NYHA: New York Heart Association; OR: Odds ratio; SCr: Serum creatine; UA: Uric acid

The included variables were also used to investigate their association with AKI-RRT or in-hospital death. Logistic regression analysis also showed that proteinuria (OR for trace to 1+ proteinuria = 3.639, 95% CI 1.99–6.64, *P* < 0.001; OR for 2–3+ proteinuria = 5.71, 95% CI 1.88–17.36, *P* = 0.002), elevated eGFR (OR 0.958, 95% CI 0.94–0.98, *P* < 0.001), complex surgery(OR 4.02, 95% CI 1.89–8.53, *P* < 0.001) and increased uric acid (OR 1.001, 95% CI 1.00–1.01, *P* = 0.044) were associated with AKI-RRT in patients with renal dysfunction. On the other hand, mild proteinuria (OR 2.59, 95% CI 1.29–5.23, *P* = 0.008) was also an independent risk factor for in-hospital death (Table 2).

### Subgroup analyses in patients with diabetes

In patients with diabetes mellitus (n = 269) (Table 3), subgroup analysis showed that mild proteinuria (trace to 1+), instead of severe proteinuria (2–3+), was a risk factor for AKI in patients with kidney dysfunction and diabetes (OR for trace to 1+ proteinuria = 1.925, 95% CI 1.028–3.602, *P* = 0.041). Meanwhile, hemoglobin (OR 0.977, 95% CI 0.961–0.993, *P* < 0.001) and albumin (OR 0.932, 95% CI 0.871–0.997, *P* = 0.04) are protective factors of AKI in these patients (Table 4).

**Table 3:** Perioperative characteristics of the subgroups of diabetes mellitus classified with proteinuria

Characteristics	Negative			Trace-1 +			2–3 +			
	Non-AKI (N = 89)	AKI (N = 84)	*p*	Non-AKI(N = 21)	AKI (N = 47)	*p*	Non-AKI (N = 3)	AKI (N = 12)	*p*	
Demographic data										
Male (%)	60 (63.2)	63 (69.2)	0.439	13 (61.9)	32 (68.1)	0.782	3 (100)	10 (83.3)	0.463	
Age (years)	66.47 ± 9.59	66.97 ± 7.36	0.695	69.1 ± 5.96	64.09 ± 9.05	0.009	68.67 ± 7.57	60.67 ± 7.29	0.115	
BMI (kg/m^2^)	23.77 ± 2.98	23.57 ± 3.21	0.675	25.32 ± 2.46	23.28 ± 2.56	0.004	22.59 ± 4.50	24.58 ± 3.77	0.536	
Comorbidities										
Hypertension (%)	68 (71.6)	72 (79.1)	0.241	15 (71.4)	33 (70.2)	0.920	3 (100)	11 (91.7)	0.617	
NYHA grade 3–4 (%)	57 (64.0)	55 (65.5)	0.875	9 (47.4)	23 (50.0)	0.848	0 (0)	6 (54.5)	0.209	
LVEF	54.71 ± 11.56	53.71 ± 13.66	0.666	59 ± 10.54	49.56 ± 11.64	0.032	54.67 ± 17.38	50.88 ± 4.58	0.743	
Baseline laboratory indices										
Hemoglobin (g/L)	127.24 ± 15.69	121.19 ± 17.19	0.014	130.71 ± 16.39	117.20 ± 20.07	0.005	127.33 ± 8.50	123.50 ± 16.34	0.709	
Albumin (g/L)	40.56 ± 3.81	39.09 ± 3.45	0.008	40.14 ± 4.27	38.17 ± 5.73	0.168	39.33 ± 3.05	39.67 ± 3.32	0.881	
eGFR (ml/min/1.73 m^2^)	48.03 ± 10.03	46.88 ± 9.78	0.433	42.90 ± 11.67	41.87 ± 12.21	0.745	25.16 ± 25.20	41.60 ± 13.02	0.125	
Surgery										
Sole valve (%)	27 (28.4)	28 (30.8)	0.694	3 (14.3)	14 (29.8)	0.172	0 (0)	1 (8.3)	0.162	
Sole CABG (%)	64 (67.4)	56 (61.5)	0.823	17 (81.0)	32 (68.1)	0.254	3 (100)	10 (83.3)	0.742	
Valve & CABG (%)	4 (4.2)	7 (7.7)	0.227	1 (4.8)	1 (2.1)	0.682	0 (0)	1 (8.3)	0.162	
CPB duration (mins)	129.83 ± 141.95	111.68 ± 46.67	0.448	116.17 ± 53.37	103.75 ± 25.65	0.432	Null	94.50 ± 26.16	null	
Cross-clamp duration (mins)	61.85 ± 22.51	83 ± 144.51	0.395	62.83 ± 15.48	54.53 ± 20.11	0.365	Null	45.33 ± 40.77	Null	
Prognosis										
In-hospital mortality (%)	0 (0)	4 (4.4)	0.055	1 (4.8)	3 (6.4)	0.794	1 (33.3)	0 (0)	0.200	
Length of hospital stay (days)	15 (11,20)	14 (11,19)	0.462	17 (12,19)	15 (12,23)	0.666	26 (quartile unavailable)	13 (10,14)	0.018	

AKI: Acute kidney injury; BMI: Body Mass Index; BUN: Blood Urea Nitrogen; CABG: Coronary artery bypass grafting; CPB: Cardiopulmonary bypass; DM: Diabetes mellitus; eGFR: Estimated glomerular filtration rate, calculated by CKD-EPI formulae; ICU: intensive care unit; LVEF: Left ventricular ejection fraction; NYHA: New York Heart Association

The values are expressed as the median (IQR) and mean ± SD or number (%)

P-values are the results of unpaired t-test or Mann–Whitney U test for continuous variables, and χ^2^ test or Fisher’s exact test for categorical variables

**Table 4:** Logistic regression of risk factors for AKI in diabetic subgroup

	Univariate analysis			Multivariate analysis		
	OR	95%CI	P value	OR	95%CI	*P* value
Baseline laboratory indices						
Hemoglobin (g/L)	0.973	0.958–0.988	< 0.001	0.977	0.961–0.993	< 0.001
Albumin (g/L)	0.907	0.850–0.967	0.003	0.932	0.871–0.997	0.04
Proteinuria						
0	1			1		
Trace-1+	2.336	1.296–4.212	0.005	1.925	1.028–3.602	0.041
2+–3+	4.176	1.141–15.283	0.031			NS

AKI: Acute kidney injury CI: Confidential interval; NS: Not Significant

## Discussion

We retrospectively collected the clinical data of 1546 patients with renal dysfunction undergoing cardiac surgery. After adjusting for variables affecting proteinuria (Additional file 1: Table S1), we found that preoperative proteinuria was an independent risk factor for postoperative AKI in patients with renal dysfunction, and the risk of AKI increased with the severity of proteinuria. Further subgroup analysis showed mild preoperative proteinuria was an independent risk factor for AKI after cardiac surgery in patients with renal dysfunction diabetes. This is the first evaluation of preoperative proteinuria in patients with eGFR < 60 mL/min/1.73 m^2^ to predict AKI and its outcome after valve or CABG surgery so far.

Previous studies have suggested that proteinuria can predict hospital-acquired AKI. Huang et al. included 1051 patients undergoing CABG surgery [7], and the results showed that preoperative mild (trace to 1+) and severe (2+–4+) proteinuria were independent risk factors for postoperative AKI, which was consistent with our results. Li et al. included patients with eGFR greater than 60 mL/min/1.73 m^2^ undergoing cardiopulmonary bypass [8]. The result showed a graded increase in the hazards of AKI along the worsening proteinuria severity, which was similar to our study.

Proteinuria increases the incidence of AKI after cardiac surgery in patients with renal dysfunction through multiple mechanisms. First of all, proteinuria is not only the marker of glomerular and renal tubular epithelial cell damage but is also involved in endothelial dysfunction [11]. Therefore, patients with proteinuria can barely tolerate hemodynamic changes due to systemic endothelial dysfunction, especially in patients with eGFR less than 60 mL/min/1.73 m^2^. On the other hand, tubular reabsorbing urinary protein triggers the expression of a series of pro-inflammatory molecules, such as monocyte chemotaxis protein-1, osteopontin, and endothelin-1, which lead to renal cell proliferation, macrophage and monocyte activation, matrix deposition, eventually lead to tubulointerstitial damage [12, 13]. Chronic proteinuria can cause tubulointerstitial inflammation and fibrosis, further impairing renal reserve capacity in patients with renal dysfunction and reducing tolerance to hemodynamic changes and nephrotoxic insults. During cardiac surgery, CPB, ischemia–reperfusion injury, endotoxemia, and surgical trauma lead to a pro-inflammatory state that is more prone to ischemic injury [14, 15].

Statistical analysis was performed on underlying disease of patients in each group (Table 1), but underlying disease was not significantly associated with AKI in logistic regression. In China, there is a high proportion of valve surgeries, including numerous double or triple valve surgeries. Since a patient may have multiple underlying diseases and be treated for multiple diseases in a single episode of cardiac surgery, we also investigated the relationship between surgical types and AKI. We found that valve & CABG was associated with AKI requiring RRT. Table 2 showed that valve & CABG were associated with AKI and in-hospital death in univariate analysis, but multivariate regression failed to show the association between valve & CABG and AKI. One of the possible reasons is that other factors in the multivariate analysis were more strongly associated with AKI and in-hospital death.

In the subgroup analysis of renal dysfunction with diabetes, even mild proteinuria was an independent risk factor for AKI after cardiac surgery. Of note, severe proteinuria (2–3+) was not associated with AKI. The possible reason was that the number of cases of diabetes complicated with severe proteinuria was small (N = 15). Table 3 showed that in these patients AKI was not associated with any of the included factors. Therefore, the risk of AKI in this specific population with kidney dysfunction complicated with diabetes and mild proteinuria shall be evaluated timely, and avoidance of preoperative anemia and hypoproteinemia may be a potential effective intervention. Further investigations are needed to identify the preventive role of these factors.

There are still some limitations in this study. Proteinuria is the main manifestation of diabetic nephropathy in the early stage, and the glomerular filtration rate can remain normal at this stage. However, the subgroup analysis failed to distinguish between renal dysfunction complicated by diabetes and mid-late diabetic nephropathy. Therefore, the significance of the results of this study for patients with early diabetic nephropathy needs to be further clarified. Secondly, although dipstick is cheap and convenient for routine urine testing, it is not quantifiable and may cause errors in a single test. Thirdly, this study was single-center and retrospective. The urinary albumin to creatinine ratio (ACR) was not examined preoperatively in patients undergoing surgery. Although ACR is favored for quantitative proteinuria detection, dipstick examinations remain the most convenient and inexpensive choice for screening. Therefore, we hope that this study can help surgeons pay more attention to the complement of urine examination in patients with CKD. Besides, the potential effect of the intervention of urinary protein in reducing the incidence of AKI needs to be elucidated prospectively. Fourthly, inflammatory markers were not included since inflammatory factors are not routinely tested before cardiac surgery. Whether proteinuria mediates preoperative inflammatory state and causes AKI needs further study. Finally, several potential risk factors (e.g. Intraoperative hemodynamic condition, postoperative hemodynamic state, transfusion amount, volume status etc.) were not collected in this study.

## Conclusions

Preoperative proteinuria was found to be an independent risk factor for AKI after cardiac surgery in patients with renal dysfunction, and the risk of AKI increased with the severity of proteinuria. For patients with stages 3–4 CKD, implement of preoperative routine urine examination, management of anemia in patients with proteinuria, avoidance of prolonged cross-clamp duration may be important methods to prevent AKI.

## Supplementary Information

**Additional file 1**.Variables correlated with Urinary Protein.

## Data Availability

The datasets used and/or analyzed during the current study are available from the corresponding author on reasonable request.

## Abbreviations

AKI: Acute kidney injury
AKI-RRT: Acute kidney injury requiring renal replacement therapy
BMI: Body Mass Index
BUN: Blood Urea Nitrogen
CI: Confidential interval
CABG: Coronary artery bypass grafting
CPB: Cardiopulmonary bypass
DM: Diabetes mellitus
eGFR: Estimated glomerular filtration rate, calculated by CKD-EPI formula
ICU: Intensive care unit
LVEF: Left ventricular ejection fraction
NS: Not significant
NYHA: New York Heart Association
OR: Odds ratio
SCr: Serum creatine
UA: Uric acid
