# Supplementary material for: Proteinuria is a risk factor for acute kidney injury after cardiac surgery in patients with stages 3–4 chronic kidney disease: a case control study
Source: BMC Cardiovasc Disord. 2023 Feb 10;23:77. doi: 10.1186/s12872-023-03102-4 (PMC9912481; doi:10.1186/s12872-023-03102-4)
Supplement: Supplementary file 1 — Additional file 1.Variables correlated with Urinary Protein. [file 12872_2023_3102_MOESM1_ESM.docx]

Supplementary Table 1. Variables correlated with Urinary Protein

| Variables |  |  |  |  |  |  |  |  |
| --- | --- | --- | --- | --- | --- | --- | --- | --- |
| Continuous Variables | Age | BUN | SCr | eGFR | UA | Hgb | Alb | CKD stage |
| *r* | -0.07 | 0.14 | 0.19 | -0.22 | 0.10 | -0.10 | -0.09 | 0.20 |
| *P* | 0.006 | <0.001 | <0.001 | <0.001 | <0.001 | <0.001 | 0.001 | <0.001 |
| Categorical Variables | Male | DM |  |  |  |  |  |  |
| *P* | 0.016 | <0.001 |  |  |  |  |  |  |

The correlation between categorical variables and urinary protein was determined by unpaired t-tests.

Alb: Albumin; BUN: Blood urea nitrogen; CKD: Chronic kidney disease; DM: Diabetes mellitus; eGFR: Estimated glomerular filtration rate, calculated by CKD-EPI formulae; Hgb: Hemoglobin; SCr: Serum creatinine; UA: Uric acid.
